# Implications of Information Theory for Computational Modeling of Schizophrenia

**DOI:** 10.1162/CPSY_a_00004

**Published:** 2017-10-01

**Authors:** Steven M. Silverstein, Michael Wibral, William A. Phillips

**Affiliations:** 1 Division of Schizophrenia Research, Rutgers University, Piscataway, New Jersey; 2 MEG Unit, Brain Imaging Center, Goethe University, Frankfurt, Germany; 3 School of Natural Sciences, University of Stirling, Stirling, UK

**Keywords:** schizophrenia, psychosis, negative symptoms, information theory, perception, cognition, computational modeling, infomax, synergy, predictive coding, cognitive control

## Abstract

Information theory provides a formal framework within which information processing and its disorders can be described. However, information theory has rarely been applied to modeling aspects of the cognitive neuroscience of schizophrenia. The goal of this article is to highlight the benefits of an approach based on information theory, including its recent extensions, for understanding several disrupted neural goal functions as well as related cognitive and symptomatic phenomena in schizophrenia. We begin by demonstrating that foundational concepts from information theory—such as Shannon information, entropy, data compression, block coding, and strategies to increase the signal-to-noise ratio—can be used to provide novel understandings of cognitive impairments in schizophrenia and metrics to evaluate their integrity. We then describe more recent developments in information theory, including the concepts of infomax, coherent infomax, and coding with synergy, to demonstrate how these can be used to develop computational models of schizophrenia-related failures in the tuning of sensory neurons, gain control, perceptual organization, thought organization, selective attention, context processing, predictive coding, and cognitive control. Throughout, we demonstrate how disordered mechanisms may explain both perceptual/cognitive changes and symptom emergence in schizophrenia. Finally, we demonstrate that there is consistency between some information-theoretic concepts and recent discoveries in neurobiology, especially involving the existence of distinct sites for the accumulation of driving input and contextual information prior to their interaction. This convergence can be used to guide future theory, experiment, and treatment development.

## INTRODUCTION

Schizophrenia is a disabling psychiatric disorder that is characterized by perceptual distortions, hallucinations, delusions, disorganized thinking, bizarre behavior, loss of motivation, and declines in role functioning. It typically has an onset in late adolescence or early adulthood, and it is associated with significant medical comorbidities (e.g., hypertension, diabetes, inflammation); reduced lifespan; marked impairments in educational, vocational, and social functioning; high rates of unemployment; high treatment costs; and psychological and economic burdens for families. Despite the accumulation of massive amounts of research findings, however, treatment outcomes and lifespan have not improved significantly, as has been the case with many other illnesses (e.g., cancer, heart disease; Insel, 2010). One reason for the lack of progress is the lack of a cohesive theoretical framework within which to understand the available data. Due to this, it has been proposed that computational modeling could be useful for clarifying the core biobehavioral processes inherent to the disorder (Silverstein, Moghaddam, & Wykes, 2014).

The goal of computational psychiatry is to provide a bridge between findings from neuroscience and our understanding of macro-level mental dysfunctions and behaviors (Montague, Dolan, Friston, & Dayan, 2012). This emerging field is focused on clarifying, via computational models, the nature of the brain’s work (Phillips & Singer, 1997), as opposed to merely describing the regions or patterns of brain activity that are correlated with cognitive and behavioral functions. Computational models formally describe neural processes in terms of mathematical relationships (Friston, Stephan, Montague, & Dolan, 2014), which allows for the effects of multiple hypothesized parameters and their interactions to be rapidly tested. Through these efforts, it is hoped that a fuller understanding of the molecular, cellular, and microcircuit bases of altered cognitive and behavioral phenomena will be gained (Adams, Huys, & Roiser, 2016). This is critical, because it has been argued that, at present, we do not fully understand these bases for even a single symptom of a single psychiatric disorder (Wang & Krystal, 2014). By generating and rapidly testing hypotheses ex vivo for their likely validity, we may also hope to gain, on average, both a greater yield from follow-up in vivo experimental studies and a reduced time from discovery, on the one hand, to clinical interventions, on the other.

The primary goal of this article is to highlight an approach based on information theory, including its recent extensions, for understanding several disrupted neural goal functions and the related behavioral phenomena in schizophrenia. This viewpoint has not been discussed in recent reviews of computational psychiatry, but it may be as powerful as the prevailing com putational approaches to schizophrenia, and it is more centered than they are on impaired neural information-processing capabilities. The text here is divided into five parts. Each of these covers a different set of concepts from information theory, although the concepts from earlier sections are necessary for understanding the later sections. Moreover, the sections are arranged so that, as they progress, each deals with an increasingly complex aspect of schizophrenia. In the following two sections, we present the basic concepts of information theory and demonstrate their relevance to reconceptualizing several phenomena in schizophrenia, including slowness of processing, reduced attentional capacity, and reduced sensory gating. In the third section, we discuss the concept of infomax and how this is relevant to understanding increased stimulus intensity and broadened sensory tuning in schizophrenia. In the fourth section, we present the concept of coherent infomax and describe its utility for explaining failures in perceptual organization, thought organization, context processing, selective attention, and lexical disambiguation, as well as disorganized symptoms, in schizophrenia. The fifth section covers a recent extension of information theory called *partial information decomposition*. This framework is described, and we discuss its relevance to understanding the normal operation of two neural goal functions (*coding with synergy* and *predictive coding*) and cognitive control, as well as their impairments in schizophrenia. A final section provides a brief summary of the major themes of the article and highlights several additional issues regarding the application of information theory to understanding schizophrenia, including a comparison to other computational models that have been useful for studying the disorder. Throughout the discussion, every effort is made to emphasize the concepts and to minimize the use of mathematical detail, which can be found in the original articles cited. One of our goals is to have this article serve as an introduction to information theory for schizophrenia researchers who might otherwise not be familiar with this perspective.

## INFORMATION THEORY BASICS

*Information theory*, or *communication theory* as it was originally called, was developed by Shannon (1948), who was influenced by the prior work of Nyquist (1924) and Hartley (1928) on the issues affecting message communication in telegraphy. However, information theory concepts can be used to understand any system wherein messages are sent from one place to another (Campbell, 1982). In this theory, *information* does not refer to the meaning of a message, but rather to the degree to which the message reduces uncertainty regarding the state of the sender or the world. A key concept in information theory is *Shannon information*, which is a measure of the extent to which the possibilities for future states of the world are constrained after one receives a message or signal. For example, think of receiving a message, one letter at a time, and that message starts with the letter “D.” Having read that first letter, the universe of possible messages has now been reduced from all possible messages to only those that start with the letter “D.” Stated differently, reading the first letter has reduced the uncertainty (i.e., reduced the remaining possibilities) about the message one is receiving. The extent of reduction in uncertainty provided by information received up to a given point in time can be measured by the uncertainty before receiving the message divided by the uncertainty afterward, as measured by probabilities *p*(.) [e.g., 1/*p*(*D*)]. The value of Shannon information is obtained by deriving the logarithm of the above quantity: *h*(*D*) = log [1/*p*(*D*)]. This logarithm is formulated in base 2 by convention, and this gives the Shannon information in bits (see below). The average of the Shannon information over all possible outcomes (e.g., all possible first letters of the message) is the *Shannon entropy* of a variable (e.g., the first letter of the message), referred to simply as the *entropy* from here on.

Entropy has some highly intuitive properties. For example, for equiprobable messages or signals, the Shannon information of a specific signal is just the number of yes/no questions that would have to be asked before the value of the signal can be arrived at. For example, if the message is communicating the outcome of a fair coin toss, there are two equiprobable values, so the information can be communicated as the answer to one yes/no question, or one binary digit, called a *bit*. If there are four equiprobable values of the signal that is communicated, then two bits are necessary to convey the outcome. With eight equiprobable values, three bits are required; with 16 possible equiprobable values, four bits are required; and so on. Thus, the number of bits required to communicate one of *N* equiprobable values is the log base 2 of that number *N* of values (i.e., the exponent to which 2 must be raised to achieve that value). For cases with *N* possible outcomes, all with probability 1/*N*, the entropy is

$$H=-\sum_{i=1}^{N}\frac{1}{N}\;\cdot \;\log_{2}\left(\frac{1}{N}\right)=-N\;\cdot \;\frac{1}{N}\;\cdot \;\log_{2}\left(\frac{1}{N}\right)=\log_{2}\left(N\right)\text{.}$$

In the case of a fair coin toss, this is

$$H=-\left[\left(\text{½}\cdot \log_{2\;}\text{½}\right)+\left(\text{½}\cdot \log_{2\;}\text{½}\right)\right]\;=-\left[\left(\text{½}\cdot -1\right)\;+\left(\text{½}\cdot -1\right)\right]\;=1\;\mathrm{bit}\;\mathrm{per}\;\mathrm{toss}\text{,}$$

as we noted above.

If the outcomes are not equiprobable, the entropy is a function of the respective probabilities of the individual outcomes. For example, if we consider a coin that is built to come up ¼ heads and ¾ tails, we obtain *H* = −[(¼ log_2_ ¼) + (¾ log_2_ ¾)] = −[(¼ · −2) + (¾ · −0.415)] = 0.811 bit per toss. This example shows that less Shannon information is available on average here than for the case of equiprobable outcomes. In other words, this means that the outcomes of the biased coin toss are more predictable, so fewer bits are needed to encode them. In general, we can encode messages using fewer bits by communicating more frequent outcomes with short bit sequences, while allowing longer sequences for the rare outcomes. Entropy can thus be understood as the limit of compression achievable (using the above method or others), and therefore the “true” incompressible information content. If one tries to communicate with fewer bits than are dictated by this limit, errors are certain to arise.

Although these examples may seem far from schizophrenia, they are relevant in terms of understanding the information-processing challenges experienced by many patients with this disorder. For example, the more statistically regular the driving input (whether from sensory regions or memory), the smaller the number of likely values for the next signal (given the previous one), thereby reducing entropy, channel capacity requirements, and processing speed requirements (the latter two of which are also key information theory concepts). An example of how entropy goes down as probabilities become less random (i.e., more determined) can be seen in the sequence given by *x*_*n*__+1_ = *x*_*n*_ + 2 (e.g., 2, 4, 6, 8, … *X*, when starting with *x*_0_ = 2). In this case, the entropy associated with *x*_*n*_ given *x*_*n*__−1_ is far less than if the sequence was one in which values were drawn at random from the natural numbers at each step, such as 3, 17, 12, 61, … *X*. Similarly, with the phrase “How are you ________,” the final word is highly likely to be “doing” or “today,” rather than a word chosen at random from the English lexicon. An implication of this for schizophrenia is that the reduced exploitation of available statistical regularities that has been observed in this disorder for processing in multiple cognitive domains (e.g., perception, language, learning; Brown & Kuperberg, 2015; Todd, Michie, Schall, Ward, & Catts, 2011; Weiler, Bellebaum, Brune, Juckel, & Daum, 2009) will lead to missed opportunities for compression down to the true entropy. This will impair processing efficiency and effectiveness, and likely will lead to a subjective experience of being overwhelmed by processing requirements. This would also increase the probability of errors (in stimulus identification and the assessment of meaning) and increase the probability of statistically rare mental representations being generated.^1^ This scenario is also relevant to the findings of widespread context-processing deficits in schizophrenia (Cohen, Barch, Carter, & Servan-Schreiber, 1999; Cohen & Servan-Schreiber, 1992; Phillips, Clark, & Silverstein, 2015; Phillips & Silverstein, 2003). This is because a consequence of reduced context processing is a reduction in the abil ity to decrease uncertainty in incoming signals by exploiting their statistical dependencies on context, or, again, an increase in the number of possibilities that must be considered (i.e., processed) at any one time. Because schizophrenia can also be viewed as being characterized by noisy processing channels (see below) as well as alterations in arousal level (and intense emotions) (de Lecea, Carter, & Adamantidis, 2012), the increase in processing demands adds a significant burden to an already overly taxed system.

The maximization of efficiency in encoding as a function of signal probability can be achieved via several techniques. Here we demonstrate this effect with one well-known method, although we note that it is not known what data compression algorithms are used by the brain. Because it is generally agreed that data compression does take place, however, we believe it is useful to consider the consequences of data compression failure for schizophrenia. A classic example of data compression can be seen using the technique known as *Huffman coding* (Huffman, 1952). This example involves the number of bits used to code the letters of the alphabet plus blank space. Rather than encoding every letter as if it had an equal probability of occurring, it is more efficient, as we noted above, to encode the most frequently occurring value (“blank space”) with the shortest code, and then to use increasingly longer codes for letters with smaller and smaller probabilities. This is indeed very similar to what is done in Morse code, in which the symbol for the letter “e” (which is the *letter* most fre quently used in the English language) is one dot (followed by a pause in signaling that also consumes capacity). Different versions of Huffman coding of the English alphabet have been proposed, based on slightly different frequency calculations of each letter, and on whether a code for a blank space was included. In an example of a code using a blank space, the blank is encoded as 01, and “e” is encoded as 1100. Other frequently occurring letters, such as “t” (1111), “a” (0000), and “i” (1001), can also be encoded using four bits. However, letters that occur with decreasing frequency, such as “g” (001001), “k” (1010000), “v” (11010001), “q” (110100001), and “j” (1101000000), are encoded using six, seven, eight, nine, or ten bits, respectively (MacKay, 2003). It can be shown that coding using this strategy leads to a reduced processing requirement (i.e., fewer bits per message, on average) relative to when each possible value is encoded on the basis of its proportion of the total values. That is, as in the example of the fair and unfair coin tosses described earlier, the average number of bits required to process English letters during reading is less if Huffman coding is used than if each letter were represented as having a 1/26 probability. In the latter case, six letters can be coded with four bits, and the other 20 letters coded in five bits, for an average of 4.7 bits per letter. With Huffman coding, in the examples above, the most common letters or symbols can be coded in four bits, and the average number of bits per letter is 4.15, which represents a 12% improvement in efficiency (and a corresponding reduction in processing demands; MacKay, 2003). Although the existence of Huffman coding in biological neural networks has not been demonstrated, such coding has been used to model aspects of cognition, such as memory function (Boguslawski, Gripon, Seguin, & Heitzmann, 2014). We believe, therefore, that it would be useful to determine the extent to which this and other data compression algorithms approximate the processing characteristics of healthy subjects, and the extent to which perturbations in these algorithms generate data that approximate what is observed in people with schizophrenia. Information theory provides a basis for formally expressing data compression mechanisms, for facilitating their translation to modeling and experimental studies, and for understanding the consequences of a breakdown in these mechanisms. A major point here is that the previously mentioned impairment in detecting and representing probabilistic relationships in schizophrenia would be expected to have the effect of increasing encoding requirements, which would, among other consequences, impose a burden on attention, working memory, and other cognitive processes.

It is known from much prior work on information theory that communication requirements can be reduced by encoding information in longer units (i.e., block coding). This reduces the uncertainty within and between message units by creating relatively few highly frequent, or “typical,” units, in comparison to an overwhelmingly large number of extremely infrequent units. It can be seen, therefore, that an important effect of the reduced ability to bind information into larger units, as is found in perception, attention, working memory, and language in schizophrenia (Haenschel et al., 2007; Phillips, Clark, & Silverstein, 2015; Phillips & Silverstein, 2003; Silverstein & Keane, 2011; Uhlhaas & Silverstein, 2005; see also the Coherent Infomax section below), is that the demands on information transmission will be increased (i.e., increased further above the demands of reduced probabilistic effects, as noted above). This would lead to the requirement for greater downstream processing capacity and enhanced processing speed to maintain adequate adaptation. Because people with schizophrenia do not have superior processing capacity and processing speed, they will often appear to be characterized by *reduced* processing capacity and slower information processing, as has been demonstrated many times (Leonard et al., 2013; Nuechterlein & Dawson, 1984).

An important aspect of the argument above is that characterizing schizophrenia in terms of deficits alone (e.g., reduced processing capacity, slowed processing, or broadened neuronal tuning—see below) may provide an incomplete view of the disorder. Rather, the impaired ability to keep internal entropy at normal levels that is caused by reduced sensitivity to probabilistic relationships, reduced grouping of mental representations, and reduced context processing leads to increased processing demands, which may be interpreted erroneously as primary capacity or processing speed limitations. Clarifying the extent to which capacity and speed are reduced as a primary effect of the illness versus that to which increased demands overwhelm available capacity is an important question for future research, and one in which information theory metrics may have practical utility in terms of generating a reliable and valid biomarker.

We can think of at least three ways that research can progress in this direction. First is that tasks and experiments can be designed to manipulate probabilistic relationships in the stimulus set, and the data can be quantified in terms of entropy and Shannon information to determine the sensitivity of patients to these manipulations and any changes in this sensitivity in response to treatment. Second, data can be modeled to determine the extent to which compression is being used (and via which algorithms), and patients and controls can be compared on this metric and/or patient change over time can be assessed. Third, experimental data can be analyzed using newer statistical techniques that are based on information theory, such as the maximal information coefficient (MIC; Reshef et al., 2011), which quantifies the level of association and overlap between variables, regardless of whether these relationships are linear or nonlinear.^2^ Metrics such as the MIC, which involve the concept of mutual information—or the amount of Shannon information that can be obtained about one variable by knowing about a second variable—are especially useful for studies quantifying the effects of spatial, temporal, and semantic contexts on neural and behavioral responses and the impairments in such processing caused by schizophrenia. The MIC can also be used to examine connectivity patterns within brain activity from fMRI studies (Zhang, Sun, Yi, Wu, & Ding, 2015), which can help determine whether both behavior and neural activity fit information-theory-derived hypotheses. Mutual information, and information theory metrics in general, have already demonstrated their utility for modeling multiple aspects of functioning that are impaired in schizophrenia, including perception (Zhaoping, 2014) and cognitive control (Fan, 2014; see below).

## APPLICATIONS OF INFORMATION THEORY TO NEURAL SYSTEMS

Information processing in neural systems is conceptually constrained by the fact that (almost all) neurons have a clear distinction between their inputs, registered at synapses located on dendrites, and outputs, sent via their axons. In other words, information passes through a neuron in one direction only. We will therefore discuss neural information processing in terms of local processors that take inputs and produce outputs. In passing through such a processor, the total information from the inputs may be fully relayed, when the output bandwidth allows for this, or (in nearly all cases) reduced, when the output bandwidth is smaller than that of the inputs considered jointly. In the latter case, the output information may be selected to come from one of the inputs more than from others, or it may be information that is provided by several inputs coherently, or it may be a synthesis of the input information that can only be understood when considering all relevant inputs together (see Wibral, Priesemann, Kay, Lizier, & Phillips, 2015). All of these types of output information can also coexist simultaneously if there is enough output bandwidth. These operations on the input information can be formally expressed in terms of various neural goal functions, which will be described in increasing order of complexity in each of the following sections. Importantly, due to the potentially noisy operation of biological neural processors, a part of the output information may not come from the inputs at all; that is, it may be considered noise generated within the processor itself, and this factor must also be taken into account when formally modeling neural processing.

Our first, and most basic, application of information theory to schizophrenia involves a consideration of information transmission through a noisy neural processor, without addressing how inputs specifically contribute to the outputs. To improve accuracy regarding information transmission through a noisy channel, various strategies for encoding or representing information have been devised. In several cases these strategies are relevant to schizophrenia, since this disorder has long been considered to be characterized by excessive noise during infor mation processing (Christensen, Spencer, King, Sekuler, & Bennett, 2013; Spitzer & Neumann, 1996). There are likely to be several sources of excessive neuronal noise in schizophrenia, in cluding excessive background (i.e., stimulus-independent) synchronization (Silverstein, All, et al., 2012) and other forms of hyperconnectivity (Anticevic et al., 2015) in cortical processing; reduced increases in synchronization during the processing of relevant stimuli (Uhlhaas & Singer, 2010); and cortical hypodopaminergia, leading to a greater-than-normal spread of neural activation (e.g., excessive activation within semantic networks corresponding to thought disorder), as opposed to the more typical, focused zones of activation (Spitzer & Neumann, 1996). The relative effects of noise can also be magnified in schizophrenia if the signals are weaker than normal. The latter effect has been proposed to occur in visual processing in schizophrenia, due either to a loss of retinal ganglion cells and/or their axons that compose the optic nerve, as measured by optical coherence tomography, or to weaker photoreceptor, bipolar, and ganglion cell firing, as measured by electroretinography (Celik et al., 2016; reviewed in Silverstein & Rosen, 2015). Reduced signal can also result from a loss of neurons in visual cortex, which has been observed in schizophrenia but not in bipolar disorder (Mitelman & Buchsbaum, 2007; Reavis et al., 2017). It is also possible, of course, that both weaker signaling and excessive baseline noise are present in the disorder.

One way in which information transmission can be improved in an intrinsically noisy processor is to increase the intensity of the signal (i.e., to increase the signal-to-noise ratio). This raises the intriguing possibility that the hyperintense perceptual experiences often found in schizophrenia (Bunney et al., 1999; Chapman, 1966; Klosterkotter, Hellmich, Steinmeyer, & Schultze-Lutter, 2001; McGhie & Chapman, 1961), especially early in the course of the illness, could represent a compensatory response to increased noise. To our knowledge, this hypothesis has never been examined, and the consensus opinion appears to be that increases in perceived stimulus intensity, as well as reduced sensory gating, are primary phenomena in schizophrenia (Rapin et al., 2012; Swerdlow & Geyer, 1998). However, this issue warrants further investigation because, if the increased signal intensity in schizophrenia is indeed compensatory, this would suggest that treatments could be developed to intervene at this level (i.e., in addition to reducing increased noise, reducing compensatory signal intensification could be viewed as a separate treatment target).

A second way to preserve the nature of the signal in a noisy channel is to increase the redundancy in the output in order to reduce errors in signal interpretation. However, in cases in which output redundancy is excessive, the richness of content is reduced; that is, the rate of Shannon information transmission is reduced. Such a compensatory mechanism might be involved in poverty of content, superficiality, and perseveration, all of which are common aspects of thought disturbance in schizophrenia. Finally, extreme manifestations of reduced activity, such as alogia, psychomotor retardation, and catatonia, may also represent compensatory responses to increased noise in schizophrenia. If this is true, then these and other negative symptoms may be best reconceptualized not as deficit symptoms, in the original sense of Hughlings–Jackson, as is often assumed (but see Berrios, 1985, and Sass & Parnas, 2003, for critiques of this position that are consistent with the view expressed here), but rather as adaptive attempts to reduce processing errors in the face of excessive noise.

A third method to increase the signal-to-noise ratio in a noisy channel is to increase the length of neuronal refractory periods. For example, it has been shown in computational models of visual cortex function that with longer periods between firing, the effects of noise are essentially washed out (Miikkulainen, Bednar, Choe, & Sirosh, 2005). This raises the intriguing hypothesis that what has been conceptualized as slowness of processing in schizophrenia could be due in part to compensatory efforts to isolate relevant signals.

With most of the clinical phenomena noted up to this point, it is not possible to determine whether the hypothesized information-theory-derived mechanisms should be considered as explanatory or merely descriptive. In addition, we have noted that the hypotheses refer to compensatory processes rather than to the primary impairments. In all of these cases, however, metrics that quantify information-theory-derived concepts such as entropy, information, and mutual information could still be very useful for assessing the state of information-processing disruption in schizophrenia, as well as for use in clinical monitoring and prediction and in treatment development studies. We now turn to more recent developments in information theory and their relevance to cognition. These developments invoke mechanisms that appear to be candidates for the core primary neurobiological disturbances in schizophrenia.

## INFOMAX

Infomax is a hypothesized neural objective function whose goal is to maximize the information in the output *Y* of a processor with regard to its input *X* under the constraint of severe data reduction [*H*(*Y*) < *H*(*X*)]. In its original conceptualization, this function was used to demonstrate the effects of cells in the second layer of a network optimally preserving the information contained in the input units (Linsker, 1988). It has been used to model, among other things, the self-organization of receptive fields (Linsker, 1988), and has become a standard preprocessing step in machine learning (Lee, Battle, Raina, & Ng, 2006). A key focus of early work on infomax was the effects of noise. In light of the strategy of increasing redundancy as a means to reduce the effects of noise, described above, Linsker demonstrated that, in the simple case of two input cells (L) and two second-layer cells (M), when the variance in noise values (*B*) arising from the M cells themselves (i.e., not in the input) is high and the correlation or covariance (*q*) in the input between two L cells is high, then the system adapts by having adjacent M cells encode increasingly similar linear combinations of the input.^3^ Stated differently, when the processor-specific noise variance is large, M cells adapt by maximizing their activity variance, and this leads either to output redundancy (via overlapping RFs) or to overly broad neural tuning. This scenario describes what has been observed in schizophrenia, in which studies from multiple paradigms in multiple sensory domains have indicated less precise neuronal tuning than among healthy controls (Green, Lee, Wynn, & Mathis, 2011; Harvey et al., 2011; Javitt, Strous, Grochowski, Ritter, & Cowan, 1997; Rokem et al., 2011; Schallmo, Sponheim, & Olman, 2013). This adaptive strategy increases the probability that signal, and not noise, will be encoded, even if that signal is more coarsely represented than would be the case in a less noisy processor.

In the context of understanding schizophrenia, two issues raised by the equation predicting the degree of output redundancy (see footnote 3) are (1) the origin of the noise and (2) the presence of other factors that could contribute to redundancy in output processing. Regarding the former issue, we have already mentioned the effects of increased background synchrony and reduced stimulus-induced synchrony on noise levels in schizophrenia. An additional potential contributor is abnormal sensory transduction. For example, multiple studies have now indicated retinal and other ocular dysfunctions in schizophrenia and in children at risk for the disorder (Silverstein & Rosen, 2015), as well as reduced visual acuity in adult patients (Viertio et al., 2007). Regarding the latter issue, factors such as reduced cortical volume (Williams et al., 2009) and reduced dendritic branching (Moyer, Shelton, & Sweet, 2015) in schizophrenia could also contribute to coarser representations. The combination of these factors increasing noise and coarsening representations is a particularly potent setting condition for the increased output redundancy and reduced precision of perception and cognition in schizophrenia. Whatever the causes, the mathematical infomax theory developed by Linsker and others has provided a means of modeling and quantifying the extent to which schizophrenia is characterized by a reduced ability to maximize information transmission.

## COHERENT INFOMAX

The fundamental limitation of the infomax objective is that it simply seeks to transmit all the information in the input without any attempt to distinguish between the information that is currently relevant and that which is not. The theory of coherent infomax shows explicitly how this limitation can be overcome by assuming that local processors receive contextual inputs that modulate the transmission of information about the driving inputs so as to amplify the transmission of currently relevant information and suppress the transmission of irrelevant information. This contextual field (CF) input must therefore be clearly distinguished from the feedforward-driving receptive field (RF) input.^4^ CF input can arise from multiple sources, including visual information outside the classical receptive field, attentional signals, memory, and so forth. Within the original framework of coherent infomax, the entropy in an output unit, *H*(*Y*), was decomposed into four sources of information (*I*): (1) information in the output that is also in the RF but not in the CF, conceptualized by *I*(*Y* : RF |CF); (2) information in the output that is in the CF but not in the RF, or *I*(*Y* : CF |RF); (3) information in the output that is shared by both RF and CF, *I*(*Y* : RF; CF); and (4) information in the output that is in neither the RF nor the CF, *H*(*Y* |RF, CF).^5^ The neural goal function of coherent infomax is to maximize the transmission of information that is predictably related to its current context. Therefore, the weights assigned to each of the four terms listed above are positive or zero, but unequal, and can be expressed as [1 – *ε*, 0, 1, 0], with 0 < *ε* << 1; *ε* here serves to weight the goal of transmitting information that is in the RF but not in the CF slightly less than the goal of transmitting information found in both the RF and CF inputs. That is, the goal of coherent infomax is to maximize information in the RF that is predicted by the CF and, to a lesser degree, to increase the salience of novel or unique RF input, while at the same time minimizing the output effects of context that are not related to the RF input and reducing the output of information that is in neither the RF nor the CF, which can be considered to be noise. Stated differently, the goal is to evolve systems in which the global output entropy is large, while transmitting coherently related subsets of the input information. The existence of processing mechanisms that meet the criteria of coherent infomax is supported by computational, psychophysical, and neurobiological studies (Kay & Phillips, 2011; Phillips & Singer, 1997). For example, psychophysical studies of vision have indicated that the detection of autocorrelation (e.g., in element orientation) in arrays of visual features is a method by which second-order structure and shape information is detected (Barlow & Berry, 2011).

We have previously reviewed much evidence suggesting that coherent infomax is involved in a wide range of perceptual and cognitive processes in which coherent sets of information must be detected, bound together, and segregated from other sets. These processes include figure–ground segregation, perceptual organization, selective attention, lexical disambiguation, working memory, cognitive control, and some forms of learning (Kay & Phillips, 2011; Phillips & Singer, 1997). Moreover, we have demonstrated that each of these functions is deficient in schizophrenia (Phillips et al., 2015; Phillips & Silverstein, 2003). For example, over 50 studies of schizophrenia have demonstrated impaired perceptual organization, or the ability to group separate elements that belong to a shape or contour into a unified percept (Silverstein, 2016; Silverstein, All, et al., 2012; Silverstein et al., 2009; Silverstein & Keane, 2011; Uhlhaas & Silverstein, 2005). Because normal perceptual organization involves increasing the salience of elements that are predictably related to their context, the impairments of perceptual organization found in schizophrenia provide strong evidence for a reduction in the effects of context on processing.

Formal thought disorder (e.g., fragmentation in thinking and loose associations) has also been interpreted as a weakening of the normal predictive constraints that words or ideas have on the activation of subsequent words and ideas (Spitzer, 2000; Spitzer, Beuckers, Beyer, Maier, & Hermle, 1994). Moreover, multiple studies (reviewed in Phillips & Silverstein, 2003; Silverstein & Keane, 2011; Uhlhaas & Silverstein, 2005) have indicated that reduced organi zation of visual information is significantly related to reduced thought organization in schizo phrenia, supporting the hypothesis of a shared basis for these illness-related features.

Although the goal of discovering relationships is important for both learning and predic tion, the signaling of relationships that are weak would not be adaptive. That is, if the thresh old for signaling a relationship is too low, the normally dominant responses to stimuli will be given less prominence than other potential response options, and normally weak responses will become more likely to enter consciousness, function as context, and guide behavior. An example of this relative equalization of all possible contexts, as it occurs in schizophrenia, was reported by a patient who described how objects had begun to seem unconnected to their environmental contexts, and therefore meaningless. At the same time, he noted that “out of these perceptions came the absolute awareness that my ability to see connections had been multiplied many times over” (Matussek, 1952, 1987). Psychological models based on learning theory have used this idea of a reduction in the range of signal strength, corresponding to the continuum from nondominant to dominant responses, to explain disorganized behavior in schizophrenia (Spaulding, Storms, Goodrich, & Sullivan, 1986; Storms & Broen, 1969). We suggest, therefore, that it would be useful to operationalize dependent variables in studies of perception and cognition in schizophrenia in terms of coherent infomax. One way this could be done would be to use the MIC to assess the overlap between RF and CF inputs, on the one hand, and the output information (e.g., behavior, neural activation), on the other. Doing so could further advance our understanding of multiple but theoretically related aspects of schizophrenia, especially those involving reduced organization within and between mental representations.

Earlier work on the biological basis of the coherent infomax mechanism emphasized the role of N-methyl-D-aspartate (NMDA) receptors in implementing the modulatory effects of context on driving input (Phillips & Singer, 1997) and in impairment of this process in schizophrenia (Phillips & Silverstein, 2003). A problem with this view is that coherent infomax assumes that CF information and RF information come from *different* sources and that they are *integrated separately* prior to their interaction (Kay & Phillips, 2011). However, NMDA receptors are colocalized with *α*-amino-3-hydroxy-5-methyl-4-isoxazolepropionic acid (AMPA) receptors (involved in driving input), so these requirements cannot be met (Phillips et al., 2015). A first step out of this impasse was provided by a network model that unconfounded syn aptic plasticity and neuronal activation, and that assumed two sites of synaptic integration, with each site responsible for one of these two roles (Kording & Konig, 2000). Both this model and coherent infomax are supported by recent discoveries in neurobiology. For example, it is now known that neocortical pyramidal cells have separate integration sites for input of driving versus contextual information. Specifically, pyramidal cells in Layers 3 and 5 receive feed forward RF input via their basal dendrites in those layers, but modulatory CF input via their apical dendrites in Layer 1 (Larkum, 2013; Larkum, Nevian, Sandler, Polsky, & Schiller, 2009; Larkum, Zhu, & Sakmann, 1999; Phillips, 2017). Depolarizing inputs to the apical dendrites amplify the effects of driving (RF) input to the cell; hyperpolarizing inputs produce disamplification (Phillips, 2017). In cortical Layer 5 pyramidal cells, repeated driving input to the cell soma leads to a backpropagation of sodium, which increases the likelihood that calcium spikes will be initiated in the apical dendrite (leading to plasticity), a situation that is most likely to occur when the apical (contextual) and somatic (driving) inputs are correlated in terms of timing and/or the content being signaled (Larkum, 2013; Larkum et al., 2009; Larkum et al., 1999). As a result of this backpropagation-activated calcium spike (BAC) firing, the frequency of action potentials is increased and burst firing is more likely to occur, both of which are important in signaling the salience of stimuli or sets of stimuli (e.g., as in contour integration). Moreover, contextual inputs to the apical dendrite are initially kept segregated through the action of potassium channels (Harnett, Xu, Magee, & Williams, 2013; Hoffman, 2013) and then are integrated at the apical dendritic trunk. These data demonstrate the neurobiological plausi bility of coherent infomax as a goal for neocortex. They further point toward means by which top-down, lateral, and bottom-up signals may interact in vision and other modalities (Brosch & Neumann, 2014; Gilbert & Sigman, 2007; Muckli et al., 2015; Piech, Li, Reeke, & Gilbert, 2013). Evidence also suggests that the mechanisms of apical amplification and disamplification are themselves influenced by factors such as whether stimuli are attended to (Li, Piech, & Gilbert, 2008), and perhaps also by arousal level (Larkum & Phillips, 2016), two functions that are often impaired in people with schizophrenia.

Finally, we reemphasize the point made earlier that a reduction in the signaling of true relationships between stimuli and an increase (especially in early schizophrenia) in the signaling of coherence among weakly related stimuli would be expected to increase the entropy associated with any single mental representation (e.g., visual feature, word, etc.). This would occur because the number of likely associations to that stimulus in the current context would increase, thereby loosening constraints on the perceived nature of, and the response requirements associated with, that stimulus. As we noted above, this is likely to have several effects, including massively increasing the processing demands for any given stimulus, slowing processing, increasing distractibility, increasing the likelihood that an irrelevant stimulus or association would be paired with relevant information, and generating behavior that appears unrelated to the current context (i.e., bizarre or disorganized behavior).

## PARTIAL INFORMATION DECOMPOSITION

The discussion of coherent infomax above emphasized that mutual information between input and output modules can be viewed as being of one of four types: unique to the RF, unique to the CF, shared by the RF and CF, or not present in either the RF or the CF (i.e., noise). Recent developments in information theory, however, suggest that a fifth type of information must be considered. This has been termed *complementary* or *synergistic*mutual information, which can be defined as information that can be obtained only by knowing both inputs. Stated differently, this is information in the output (*Y*) that we cannot obtain by evaluating the input variables (*X*_1_, *X*_2_) separately (Wibral et al., 2015). Note that according to this view, *X*_1_ and *X*_2_ can each be single inputs or sets of inputs, and that in pyramidal cells they will always be sets composed of many inputs. Synergistic information is transmitted by “exclusive or” (XOR) decisions (see below for an example). It is also involved in coordinate transformations. The framework that includes synergistic information in its parsing of information types is known as *partial information decomposition* (PID; Bertschinger, Rauh, Olbrich, Jost, & Ay, 2014). In this framework, the output information in a neuron can be decomposed generically into a combination of information unique to each input source, information shared between the input sources, information that can only be known after evaluating both input sources, and information not in either input source, as noted above—that is,

$$H\left(Y\right)=I_{unq}\left(Y:X_{1}|X_{2}\right)+I_{unq}\left(Y:X_{2}|X_{1}\right)+I_{shd}\left(Y:X_{1};X_{2}\right)+I_{syn}\left(Y:X_{1};X_{2}\right)+H\left(Y|X_{1},X_{2}\right)\text{.}$$

Note that the term denoting shared information, *I*_shd_(*Y* : *X*_1_; *X*_2_), is the information type most heavily weighted in coherent infomax, whereas the term *I*_syn_(*Y* : *X*_1_; *X*_2_) is most important for processes dependent on synergistic information, as detailed below.

### Coding with Synergy (CWS)

Many forms of information processing are of a type for which the joint information provided by two or more inputs is essential to determine the output, whereas the information from any input alone, or a small subset of it, does not provide any information about the output. One prototypical example of this type of processing is the computation of an explicit mismatch between external input information and internal, contextual input information (e.g., predictions). If one knows only the external or the internal input, one cannot know whether or not a mismatch will arise, because this requires knowledge of the input from both sources. Thus, in this situation none of the inputs in isolation carries any information about the output, only the set considered jointly does. Coding with synergy (CWS) as a neural goal function strives to maximize the type of output information that requires considering the inputs jointly, as in the example above. This goal function is likely to be implemented at least somewhere in systems that perform processing based on internal predictions that are updated on the basis of the explicit signaling of mismatches between predictions and external inputs. CWS as a goal function can be at least approximately implemented within the neural network implementation and learning rules originally suggested for coherent infomax (Kay & Phillips, 2011; Phillips, Floreano, & Kay, 1998; Phillips & Singer, 1997), yet with a different choice of weights for the information-theoretic goal function (Wibral et al., 2015).

The effects of schizophrenia on synergistic processing are largely unknown. However, some evidence suggests that people with this disorder are characterized by problems with coordinate transformation—an operation that requires precise knowledge of both inputs in order to determine the result exactly, and that, therefore, has at least a certain amount of synergistic information between inputs and outputs. A problem with coordinate transformation in schizophrenia is suggested by increased variability and randomness, as compared to control subjects, in deliberate reaching movements (where synergistic information between perceptual information about the target location and kinesthetic information about arm/hand position is required), but not in spontaneous arm retraction after such movements (Nguyen, Majmudar, Papathomas, Silverstein, & Torres, 2016). This hypothesis is also suggested by abnormalities in eye movements in both laboratory (Lencer et al., 2015) and naturalistic (Dowiasch et al., 2014) environments, where synergistic information involving the intended location of spatial attention and eye position is required. Important questions for future research on schizophrenia are to clarify whether these examples are best explained as failures of CWS and whether other phenomena appear to be manifestations of altered CWS in schizophrenia.

### Predictive Coding

*Predictive coding* refers to the view that a basic function of the brain is to rapidly and efficiently predict the nature of recent inputs on the basis of a stored model of the world. Predictions are conveyed as top-down inputs that interact with feedforward signaling (Rao & Ballard, 1999). When there is a mismatch between input and prediction, a feedforward prediction error signal is generated that contributes to an updating of the stored world model and the generation of a more accurate prediction. A goal in such a system is to reduce prediction error signaling as much as possible. Much work by Friston, Frith, Corlett, and others (Adams, Stephan, Brown, Frith, & Friston, 2013; Corlett, Frith, & Fletcher, 2009; Corlett, Honey, & Fletcher, 2007; Corlett, Honey, Krystal, & Fletcher, 2011; Friston et al., 2014) has discussed the possibility of altered predictive coding in schizophrenia. These articles have focused primarily on the involvement of impaired predictive coding in the genesis of psychotic symptoms (e.g., hallucinations, delusions). However, the model is also useful for understanding perceptual impairments (Keane, Silverstein, Wang, & Papathomas, 2013; Wacogne, 2016), as well as smooth-pursuit eye-tracking deficits (as noted above) in schizophrenia.

From the perspective of PID, the goal of predictive coding can be viewed as that of predicting the most recently occurring input, *X*_1_(*t*), using information from a vector of inputs that have occurred in the past, **X**_**1**_**(** ***t*** **–1)**. Stated differently, the goal is to maximize the mutual information between the most recent input and prediction. In terms of the generic goal function noted in the section on coherent infomax, and substituting *X*_1_(*t*) and **X**_**1**_**(** ***t*** **–1)** for *X*_1_ and *X*_2_, respectively, the goal function for an error unit *Y* when coding prediction error can be expressed as:

(2) $$G_{PCE}$$

with the weights associated with these terms being [0, 0, 0, 0, –1]. That is, the goal function pursued in predictive coding is to minimize entropy in the error unit. More specifically, in PID, predictive coding is assumed to involve a comparison between input and a representation of a model of the world (from memory) that is processed using an XOR-like function to yield, at a neuronal-spike level, an output indicating either a match (e.g., 0) or a mismatch (i.e., a prediction error, indicating that the two input states are incompatible), which is used to update the world model. Thus, computing a prediction error amounts to a coordinate transformation (and thus a CWS-like operation, as well): inputs are transformed into prediction errors via computing their distances to predictions.

An advantage of the PID conceptualization of predictive coding is that it allows this coding to be formally compared to the infomax and coherent infomax perspectives. This formalization can help accelerate modeling of the relative contributions of impairments in each of these goal functions to aspects of schizophrenia.

### Cognitive Control

A recent information theory perspective on cognitive control (Fan, 2014) can help extend the PID model to the widely demonstrated impairment in this function in schizophrenia. In this view, the frontoparietal network, including anterior cingulate cortex (ACC) and anterior insular cortex, is not conceptualized, as it usually is, as a monitor of response conflict (Carter et al., 1998). Rather, these structures are seen as processing entropy, or uncertainty, and response conflict is viewed as a special case of increased uncertainty. If the key ideas of this model are combined with PID, this allows for cognitive control to be seen as involving a processing unit wherein potential response options are compared against stored information about the extent to which similar responses were (or were not) effective in past similar situations. This “effective-or-not” comparison can be processed using the inverse-AND (i.e., NAND)-like function to yield an output indicating either a “go” (i.e., 0, indicating that the current response option *and* the response stored in memory as being most effective in the current situational context are the same, so there is no need to generate another response) or a “no-go” (i.e., 1, generate another response option) decision. The consequences of the re sulting action are then used to update the stored model of the probabilities of success of different responses in different contexts. This reconceptualization of one of the key roles of the frontoparietal network emphasizes its importance in processing and transmitting information under conditions of uncertainty. This theory is supported by data from multiple imaging studies (reviewed in Fan, 2014) and is consistent with recent data on altered frontoparietal network activity during efforts at cognitive control in people with schizophrenia (Fornito, Yoon, Zalesky, Bullmore, & Carter, 2011). Related to the issue of uncertainty, there is evidence for increased functional connectivity between frontoparietal network nodes and sensory and default-mode network regions in schizophrenia (Tu, Lee, Chen, Li, & Su, 2013). This finding suggests that cognitive control may be further reduced by introducing statistically rare representations into the processor. This would have the effect of increasing uncertainty and the number of response options that need to be evaluated, leading to a higher risk of inappropriate responses and/or an overall slowing of responses.

## SUMMARY

The major theme of this article has been the utility of concepts from information theory to guide computational modeling of phenomena associated with schizophrenia. We began by demonstrating that basic concepts from information theory, such as Shannon information, entropy, data compression, block coding, and methods to increase signal-to-noise ratio, can be used to provide both novel understandings of cognitive impairments in schizophrenia (e.g., slowed processing, reduced attentional capacity, sensory gating) and precise quantitative metrics for use in future studies. Moreover, these insights also can help explain aspects of symptom atology in schizophrenia, thereby providing a mathematically precise basis from which to clarify symptom–cognition relationships. We then described more recent developments in information theory, such as the concepts of infomax, coherent infomax, and CWS, to dem onstrate how these can be used to develop computational models of schizophrenia-related failures in tuning of sensory neurons, noise reduction, perceptual organization, thought organization, context processing, predictive coding, and cognitive control. As with the earlier discussion of basic information theory concepts, these reconceptualizations of aspects of schizophrenia allow for precise metrics that can be used to test our hypotheses against those of other models. These hypotheses also raise many new questions that remain to be investigated. Given the current lack of understanding of the mechanisms involved in schizophrenia-related symptoms (Wang & Krystal, 2014), and given that the approach we espouse is consistent with the mechanism-driven recommendations of the NIMH Research Domain Criteria approach (Cuthbert & Insel, 2010), we suggest that this perspective warrants further consideration.

Because the biological basis of the implementation of goal functions such as coherent infomax is now starting to emerge (e.g., in the form of apical amplification), this provides a powerful framework for formal modeling of various phenomena. These findings suggest that pyramidal neurons are most realistically conceptualized as having separate sites for the accumulation of driving (RF) and contextual information (Larkum et al., 2009; Larkum & Phillips, 2016). Therefore, relevant aspects of schizophrenia can now be realistically modeled in terms of alterations in the use of these different types of information. To date, however, no formal modeling of schizophrenia-related features using this approach has been undertaken. There is also no evidence, as yet, of disrupted apical amplification in schizophrenia, although there is much evidence for altered neuronal connectivity in general (van den Heuvel, Scholtens, de Reus, & Kahn, 2015). Thus, this project, like much of computational psychiatry, is at an early stage.^6^ Nevertheless, we believe the yield from applying information-theory-based models will be great, especially given the previously demonstrated correspondence between predictions based on information-theoretic concepts and psychophysical findings in multiple percep tual and cognitive domains in schizophrenia (Phillips et al., 2015; Phillips & Silverstein, 2003).

Finally, several models have already shown success in modeling aspects of schizo phrenia. This means, at the very least, that models and modeling approaches need to be compared in order to determine which ones produce the best fits to existing data and the most useful new insights and hypotheses. In addition, there is always the possibility that some models will be more appropriate than others for certain aspects of schizophrenia. For example, impairments in reward-based learning and reversal learning have been modeled in schizophrenia (Schlagenhauf et al., 2014), and these ideas have also been used to explain anhedonia, which is a frequently observed negative symptom in the disorder (Huys, Pizzagalli, Bogdan, & Dayan, 2013). Although the information theory concepts we have emphasized can, in theory, account for learning and its impairments, thus far they have been used primarily to explain functions such as perception, selective attention, lexical disambiguation, and synchronization, as well as their impairments in schizophrenia. The utility of these concepts for understanding reward-learning deficits, anhedonia, and other negative symptoms has not yet been explored. Thus, although, as we discussed earlier in this article, our view provides a potential explanation of certain negative symptoms—such as poverty of content, perseveration, alogia, psychomotor retardation, and catatonia—it has not yet been applied to situations in which, presumably, processing of the affective valence of stimuli is altered.

Regarding what is arguably the other major modeling approach for schizophrenia, the predictive-coding model (Clark, 2013; Corlett et al., 2009; Corlett et al., 2007; Corlett et al., 2011; Friston et al., 2014), more can be said about its overlap with information theory. First, as we noted above, one of the basic principles of this model is the idea that organisms strive to achieve minimal prediction error (MPE). It has been shown that MPE is equivalent to entropy in the current stimulus representation that is not captured by the internal model of the world (Clark, 2013). However, taken to its extreme, the goal of MPE would lead organisms to simply remove themselves from all stimulation, or what has been called “the dark room dilemma” (Little & Sommer, 2013). It has therefore been noted that, rather than conceptualizing MPE in terms of conditional entropy, it is more useful to frame it in terms of maximizing mutual information between sensory input and the internal model. According to this view, a common goal of organisms is to seek out conditions in which *both* entropy and mutual information are maximized (e.g., by seeking out new experiences as long as there is enough predictability in stimulation and the outcomes of actions to allow for adaptive behavior; Little & Sommer, 2013). This goal may be adaptive, in the evolutionary sense, since it leads to continually improved adaptive fitness, or the maximization of prediction success, in a world in which complexity and change are implicitly assumed to be ongoing conditions. A view of predictive coding in which its main goal is to maximize mutual information between outcome and prediction was developed by Wibral et al. (2015) and described above in the section on partial information decomposition. Other examples of overlap between predictive coding and information theory, such as the roles of RF input and CF input in contributing to prior probability distributions and likelihood estimation, respectively, are discussed in Phillips (2012). These examples demonstrate that there are areas of overlap between predictive coding and information theory through which the approaches can mutually inform each other.

However, there are also areas where the two models are not compatible (Phillips & Silverstein 2013). For example, Phillips, Clark, & Silverstein (2015) reviewed a number of cases in which phenomena can be accounted for by the type of local interactions emphasized by the coherent infomax function, without the need to resort to top-down signaling of expectations. In addition, predictive-coding models do not provide a mechanism through which subsets of the available input could be given priority over other information during processing. In contrast, information theory concepts such as coherent infomax describe self-organizing mechanisms through which information related to the current context can be prioritized. Relatedly, predictive-coding models emphasize combining new and old information to update posterior probabilities, but they do not describe how local processors select relevant information or how multiple processing streams coordinate their activity (Phillips, 2013). As a result, at present, predictive-coding models appear best suited for explaining the development of symptoms such as delusions, but they are not easily applicable to understanding disorganized symptoms, which involve fragmentation of function. On the other hand, the approaches we have described have not yet been applied to explaining positive symptoms, so it remains to be seen whether they will add to the insights provided by predictive-coding models regarding these symptoms.

Finally, much work in schizophrenia, including applications of the predictive-coding model, has focused on top-down–bottom-up interactions. However, we believe that this framework is inadequate to account for many neural abnormalities and their effects in the disorder. Much happens locally, and via horizontal connections, and there are many reasons to expect local circuit issues with this disorder, including reduced dendritic branching. Therefore, the view presented in this article complements those involving longer-range interactions that are typically the focus of work within computational psychiatry (Friston, 2010) and the cognitive neuroscience of schizophrenia (Sheffield et al., 2015a, 2015b).

## AUTHOR CONTRIBUTIONS

S.M.S. conceived of and wrote the majority of the article. Much of the content reflects discussions over the last 20 years between S.M.S. and W.A.P. on information theory and schizophrenia. W.A.P. wrote a significant portion of the section on coherent infomax and edited and revised the manuscript as a whole. M.W. wrote much of the section on coding with synergy and edited and revised the manuscript as a whole.

## Notes

^1^ When normally rare responses occur, entropy is increased, because the range of likely values in the sub sequent input is immediately and greatly expanded.^2^ The MIC is particularly useful for understanding datasets in which the relative influences of multiple sources of influence on the output need to be determined, as with coherent infomax and coding with synergy, which are discussed in later sections of this article.^3^ *B* will increase as the noise in the M cells is increasingly uncorrelated. When *B* is high and the input from L cells is highly correlated, then the noise variance is high relative to the signal variance, and the system has to compensate in order to increase the signal strength. Specifically, when *Bq*/(1 – *q*^2^) ≥ 1, both M cells will encode the same combination of L cell activity, creating redundancy, or overly broad neural tuning. As this value decreases from 1, overlap in the M cell outputs decreases, and the tuning becomes narrower or more precise.^4^ Although we use the term *receptive field* input here and provide many examples from vision, the issues we discuss are relevant to any processor that receives afferent (RF) input as well as input from lateral connections (CF) and feedback from higher-level processors (CF).^5^ Note that in these expressions, anything to the left of a vertical bar could also be expressed as the entropy in the output [*H*(*Y*)] minus the mutual information shared between the output and everything to the right of the vertical bar [e.g., *H*(*Y*) – *I*(*Y*; *X*)]. This quantity—reflecting, in this case, entropy in the output that is independent of another variable or set of variables (e.g., RF and/or CF and/or noise)—is known as *conditional entropy*. An alternative way to view conditional entropy is that it is the amount of uncertainty in *Y* after *X* is known (Shannon, 1948).^6^ For example, the neurobiology of coding with synergy needs to be clarified, as does much of the predictive-coding model (e.g., it is not clear how/whether pyramidal cells signal prediction error).
